# A New Insight of Graphene oxide-Fe(III) Complex Photochemical Behaviors under Visible Light Irradiation

**DOI:** 10.1038/srep40711

**Published:** 2017-01-13

**Authors:** Renlan Liu, Xiaoying Zhu, Baoliang Chen

**Affiliations:** 1 Department of Environmental Science, Zhejiang University, Hangzhou, Zhejiang 310058, China; 2 Zhejiang Provincial Key Laboratory of Organic Pollutant Process and Control, Zhejiang University, China

## Abstract

Graphene oxide (GO) contains not only aromatic carbon lattice but also carboxyl groups which enhanced the aqueous solubility of GO. To study the transformation of GO nanosheets in natural environments, GO aqueous dispersion was mixed with Fe^3+^ ions to form photoactive complex. Under visible light irradiation, Fe(III) of the complex would be reduced to Fe(II) which could subsequently reduce highly toxic Cr(VI) to Cr^3+^. The electron of the reduction was contributed by the decarboxylation of carboxyl groups on GO and iron was acting as a catalyst during the photoreduction. On the other hand, the consumption of carboxyl groups may convert GO to rGO which are tend to aggregate since the decreased electrostatic repulsion and the increased π-π attraction. The formed Cr^3+^ may be electrostatically adsorbed by the rGO sheets and simultaneously precipitated with the aggregated rGO sheets, resulting the effective removal of chromium and GO nanosheets from the aqueous environment. This study may shed a light on understanding the environmental transformation of GO and guide the treatment of Cr(VI).

Graphene oxide (GO) as the most commonly used graphene derivative contains carboxylic acid at the edges and epoxy and hydroxyl groups on the basal plane, imparting a substantial degree of sp^3^ hybridization^1^, which different from sp^2^-hybridized carbon sheet of graphene with a thickness of one atom. GO nanosheets are electrically insulating^2^, aqueous dispersible^3^ and excellent adsorption ability to metal ions^4^,^5^,^6^,^7^,^8^. However, as a typical nanomaterial, GO sheets can be easily dispersed in water and may affect the aqueous environment^9^,^10^. GO also has photoelectric property because the oxygen doping opens the electron energy gap of graphene; thus pure GO could slowly be reduced by photoelectron under sunlight^11^,^12^,^13^,^14^. It was reported that GO was transformed to reduced GO (rGO) under a long time (>10 h) sunlight irradiation and air-saturated conditions. However, the adsorption of metal ions on GO may obviously affect the photochemical behavior of the nanomaterial.

Iron ions including Fe^2+^ and Fe^3+^ are common metal ions in natural environment, which may easily form complexes (denoted as Fe(II) and Fe(III)) with organic ligands^15^ including carboxylic acids such as oxalate, malonate, citrate and humic acid. More important, UV/visible light irradiation may trigger the fast reduction of Fe(III) to Fe(II) and oxidative degradation of carboxylic ligands in aerated systems^16^,^17^,^18^. This phenomenon has drawn considerable attention because of its crucial role in the carbon^19^ and iron^20^ cycles. GO is transparent and functions as a solid state Lewis acid (p*K*_a_ = 3–4) in water because of its carboxyl groups^21^,^22^,^23^,^24^. Thus, the photochemical behavior of the material combining GO with Fe^3+^ in aqueous environment is particularly interesting and has not been studied yet.

The coordinated hexavalent chromium denoted as Cr(VI) is a highly toxic and common pollutant in the aqueous environment^25^,^26^. However, the trivalent chromium ion (Cr^3+^) in a lower valent state is less toxic than Cr(VI), which can be easily adsorbed by abundant natural negatively charged particles to form coordinated trivalent chromium Cr(III) and spontaneously precipitate to leave the aqueous environment^27^. It seems reduction is a promising approach to remove Cr(VI) from the aqueous environment. It is well known that Fe(II)/Fe^2+^ is able to reduce Cr(VI) to Cr(III)^28^,^29^. Thus, the transformation of GO in a aqueous environment containing Fe^3+^ and Cr(VI) under sunlight is of crucial environmental importance but lack of study.

In this study, GO nanosheets were mixed with Fe^3+^ ions to form the GO-Fe(III) complex which was characterized with SEM, TEM, STEM, AFM, FTIR, and XPS. Subsequently, the GO-Fe(III) complex was introduced to an aqueous environment with Cr(VI) under visible light irradiation. The photoreduction of Cr(VI) to Cr(III) by GO-Fe(III) was investigated in different conditions. The characteristic differences of the GO-Fe(III) complex before and after the photoreduction were also studied. The transformation of GO in the aqueous environment with the presence of Fe^3+^ and Cr(VI) was simulated in a lab scale.

## Results and Discussion

### Characterization of GO and GO-Fe(III)

**Figure 1:**
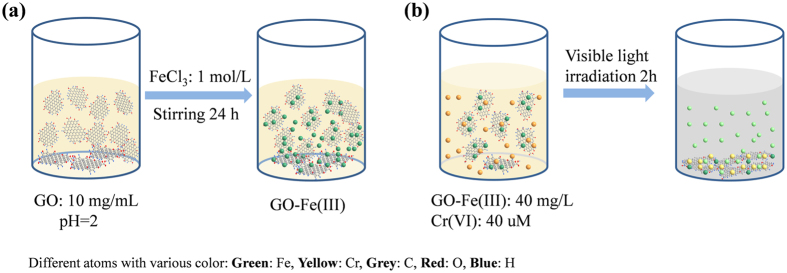
Schematic depicting (**a**) preparation of the GO-Fe(III) complex and (**b**) photoreaction after adding Cr(VI).

**Figure 2:**
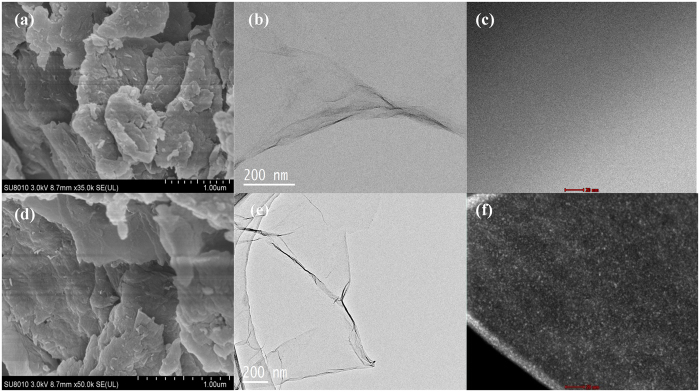
SEM (**a,d**), TEM (**b,e**), and STEM (**c,f**) images of GO (**a,b,c**) and GO-Fe(III) (**d,e,f**).

Sorption kinetics and isotherms of Fe^3+^ ions by GO were presented in Figure S1a,b, respectively. Fe^3+^ ions were fast bound by GO (Figure S1a), and the maximum saturated adsorption amounts (*Q*_max_) derived from isotherm (Figure S1b) reached 622.4 mg/g, which suggest that GO is a strong adsorbent of Fe^3+^ ions. As shown in Fig. 1a, the GO-Fe(III) complex was formed by simply mixing GO and Fe^3+^ ions at pH 2. The surface properties and microstructure of GO and GO-Fe(III) were characterized using SEM, TEM, STEM, AFM, FTIR, and XPS. The typical ripples on GO and the GO-Fe(III) complex are shown in the SEM images (Fig. 2a,d). The TEM images (Fig. 2b,e) show transparent two-dimensional nanosheet films in GO and the GO-Fe(III) materials. The surface of GO was relatively flat with slightly grooved regions on the basal planes. In comparison, the slightly aggregated loose wrinkles were observed on the surface of GO-Fe(III), which should be attributed to the cation-π interaction and Lewis acid-based interaction between GO and the Fe^3+^ ions. The STEM images (Fig. 2) show that the iron complexes were uniformly distributed on the surface of GO. The sparklet in Fig. 2f shows the coordinated Fe^3+^, and the EDX analysis also shows the peak of Fe in GO-Fe(III) (see Supplementary Figure S2). The TG analysis indicated the iron atomic ratio is approximately 1.95% in GO-Fe(III) (see Supplementary Figure S3). The structural features and thickness of the entire GO and GO-Fe(III) nanosheets were further characterized using AFM (see Supplementary Figure S4). The lateral dimensions of GO and GO-Fe(III) were several micrometers. The thicknesses of the GO and GO-Fe(III) nanosheets were approximately 1 nm and 10 nm, respectively. The microstructures of GO and GO-Fe(III) observed by AFM are consistent with those in the SEM, TEM and STEM.

**Figure 3:**
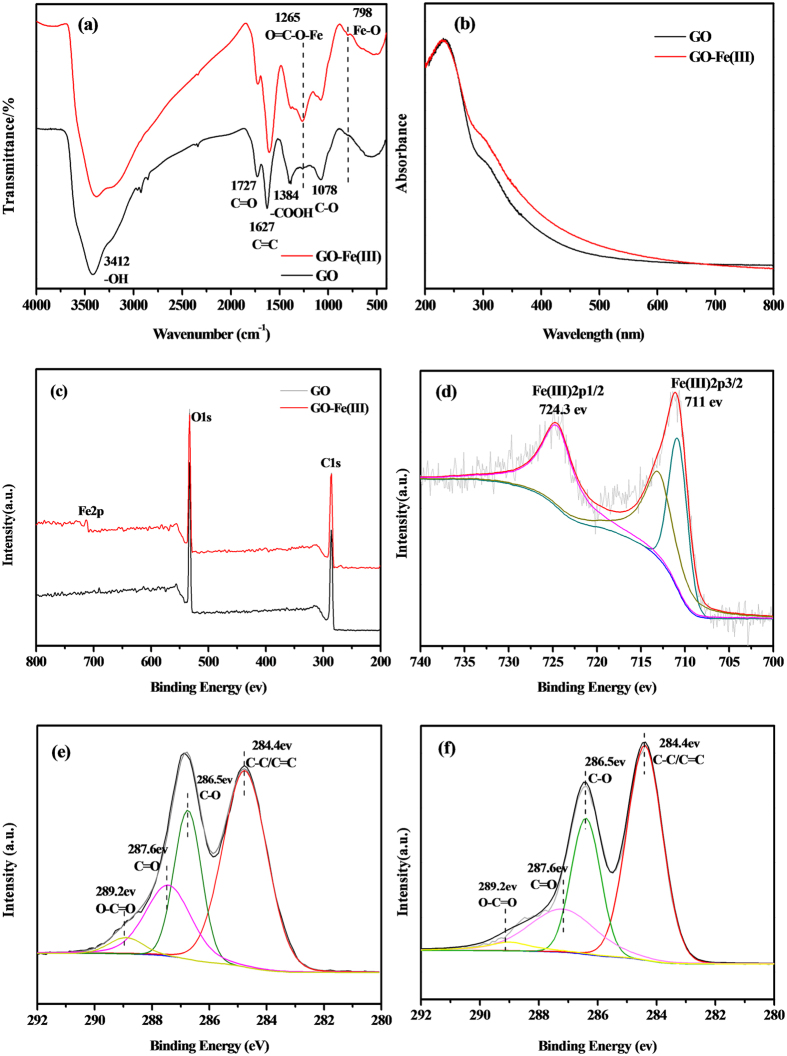
FTIR spectra (**a**) and UV-visible spectra (**b**) of the GO and GO-Fe(III) complexes. XPS spectra of GO and GO-Fe(III) at full survey (**c**), Fe2p spectra of GO-Fe(III) (**d**), C1s spectra of GO (**e**), and C1s spectra of GO-Fe(III) (**f**).

The FTIR spectra of GO and GO-Fe(III) are shown in Fig. 3a. GO contains abundant reactive oxygen functional groups, such as –COOH, –OH, C = O, and –O–30,31. Besides a strong adsorption band at 3412 cm^−1^ assigned to the –OH stretching vibration, the FTIR spectrum of GO shows sharp C = O (1727 cm^−1^), aromatic C = C (1627 cm^−1^), carboxyl O = C–O (1384 cm^−1^), alkoxy C–O (1078 cm^−1^) and epoxy C–O (1260 cm^−1^) stretching vibrations. In comparison, the band at 1627 cm^−1^, which is assigned to graphite carbon atoms ν_c=c_, did not change after the introduction of Fe^3+^. While GO-Fe(III) shows relatively strong stretching vibrations at 1265 cm^−1^, which is ascribed to the formation of O = C–O–Fe complex after introduction of Fe(III). Moreover, a new band at 798 cm^−1^ is attributed to the Fe-O group formed in GO-Fe(III). Reasonably, complexation is the primary Fe^3+^ ions-bonding mechanisms in GO. The UV-visible light absorbance spectra of GO and GO-Fe(III) are shown in Fig. 3b. GO and GO-Fe(III) have similar UV-visible adsorption profiles with the characteristic maximum at 230 nm, which is related to the π–π* plasmon, and the shoulder at approximately 300 nm is often attributed to the n–π* transitions of C = O^32^. Notably, there is increased absorbance of GO-Fe(III) from 300 to 800 nm compared with GO, mainly due to the electron transfer between oxygen containing functional groups and iron centers in the GO-Fe(III) complexes, which promotes the capacity of visible light absorption. And the red-shift of GO-Fe(III) compared with GO alone would favor the photoreaction of GO-Fe(III).

The XPS spectra of GO and GO-Fe(III) are shown in Fig. 3(c–f). The C1s and O1s peaks are observed at 285 and 532 eV, respectively (Fig. 3c). The peaks at 711 eV and 724.3 eV are assigned to Fe(III)2p_3/2_ and Fe(III)2p_1/2_, respectively, as shown in Fig. 3d, demonstrating that Fe^3+^ ions were successfully loaded onto the GO. Figure 3e shows the C1s peak of GO with a main peak at 284.4 eV, which is ascribed to the presence of C = C/C–C sp^2^ carbon atoms in the framework. The peaks at 286.5, 287.6, and 289.2 eV correlate with the carbon in C–O, the carbonyl carbon in C = O and the carboxyl carbon in O–C = O, respectively. Figure 3f shows the C1s peak of GO-Fe(III), which shows identical functional groups to those in GO. The contents of the oxygen-containing functional groups were similar for GO and GO-Fe(III), which suggests that the adsorbed Fe^3+^ ions formed sp^2^ or sp^3^ hybridized zones in GO.

### Transformation of Cr(VI) with the Existence of GO-Fe(III) under Visible Illumination

**Figure 4:**
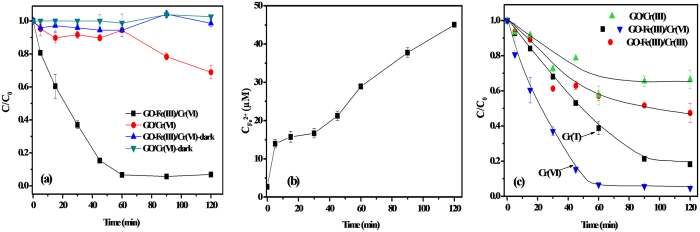
Photoreduction of Cr(VI) in the GO-Fe(III)/Cr(VI) and GO/Cr(VI) systems in the dark and under visible light irradiation (**a**). Fe^2+^ accumulation in the GO-Fe(III)/Cr(VI) system under visible light irradiation (**b**). The total Cr concentration in GO-Fe(III)/Cr(VI), GO-Fe(III)/Cr(III) and GO/Cr(VI), and Cr(VI) concentration in GO-Fe(III)/Cr(VI) under visible light irradiation (**c**). Conditions: [Cr(VI)] = 40 μM, [Cr(III)] = 40 μΜ, [GO] = 40 mg/L, [GO-Fe(III)] = 40 mg/L, pH = 3.

Figure 4a shows the time profiles of Cr(VI) reduction under visible light irradiation and dark control with 40 mg/L GO and GO-Fe(III) suspensions at pH 3. After 60 minutes irradiation, a small extent of Cr(VI) reduction occurred in the presence of GO, whereas a nearly 100% conversion rate was obtained in the presence of GO-Fe(III). In a contrast, after stirring for 30 min in the dark, the concentrations of Cr(VI) in the solution were about 35 μM and 39 μM in the system of GO-Fe(III) and GO suspensions, respectively, and then kept constant in the next 120 min under dark condition (see Supplementary Figure S5a), which indicated that Cr(VI) didn’t generate precipitates with Fe(III)^33^ and the adsorption of Cr(VI) by GO-Fe(III) was not the dominant mechanism for the removal of Cr(VI) in the solution. Moreover, these results indicate that the visible light irradiation remarkably accelerated the reduction of Cr(VI) due to the existence of the GO-Fe(III) complex. In addition, there was no noticeable reduction of Cr(VI) without illumination, demonstrating visible light irradiation is an essential reaction condition.

Fe(II) is one of the dominant reductants of Cr(VI) in the environment^34^. The production of Fe^2+^ in the GO-Fe(III)/Cr(VI) irradiation system was also detected (Fig. 4b). The amount of generated Fe(II) ions in the solution gradually increased to 45 μM after 2 h of reaction under visible irradiation. However, without irradiation, the concentration of Fe^2+^ in the solution was extremely low and kept invariant (see Supplementary Figure S1c), which further declared that the Cr(VI) reduction are mainly triggered by Fe(II) production. Figure 1b illustrated the transformation of Cr(VI) in the aqueous environment containing GO-Fe(III) under visible light irradiation.

As reported, the photolysis of Fe(III)-carboxylate complexes results in the oxidative degradation of the carboxylate ligand and reduction of the metal center to Fe(II)^17^,^18^. The rapid generation of Fe(II), as shown in Fig. 4b, should be attributed to the ligand-to-metal charge transfer (LMCT) process of Fe(III)-carboxylate complexes at the edge of GO under irradiation. The high light absorption efficiency of the GO-Fe(III) complex may also promote the photoreduction or Fe(II) generation.

The overall photoreduction process can be mainly segregated into the reduction of Fe(III) to Fe(II) and the subsequent reduction of Cr(VI) accompanied by Fe(II) oxidized to Fe(III). As shown in Fig. 4b, the rate of Fe(II) production was faster than its oxidation by Cr(VI), which is similar with the reported DOM-iron/Cr(VI) reaction system^35^,^36^. The release of Fe(II) suggested that Fe(II) formed very weak complexes with carboxylate ligand in comparison with Fe(III). After Cr(VI) was completely photoreduced to Cr(III) in 60 mins, the accumulation of Fe^2+^ ions was further increased, which indicates that the carboxyl group on the GO has a strong photochemical reactivity when it was combined with Fe^3+^. In addition, during this process, GO was partly reduced along with the carboxyl groups degraded to carbon dioxide. Without Fe(III), the photoreduction of Cr(VI) by pure GO under visible light irradiation could not occur in the first hour. However, with extended irradiation time, about 30% of Cr(VI) was photoreduced in the GO/Cr(VI) system (Fig. 4a), which may be attributed to the light responsive and photoelectric properties of GO^37^,^38^,^39^.

The change of total chromium concentration (denoted as Cr(T)) in the three reaction systems are illustrated in Fig. 4c. In the GO-Fe(III)/Cr(VI) system, the removal percentage of Cr(T) reached nearly 90% in 120 min. Moreover, the Cr(VI) concentration was approaching zero in a faster decrease rate than Cr(T) in 120 min in the GO-Fe(III)/Cr(VI) system. These results indicated that 90% of chromium elements were removed from the solution in 120 min. And the remaining chromium elements in the solution is not in the highly toxic Cr(VI) form. It is highly possible that most of the Cr(VI) complexes were photoreduced to the positively charged Cr^3+^ ions which were adsorbed by the generated rGO followed with precipitation to leave the aqueous environment. The quantitative contribution of adsorption of generated Cr^3+^ to the removal percentage of Cr(T) by GO-Fe(III) was shown in Figure S5b, suggesting that adsorption contribution of Cr^3+^ increased with the light irradiation time.

### Characterization of GO-Fe(III) after Photoreaction

**Figure 5:**
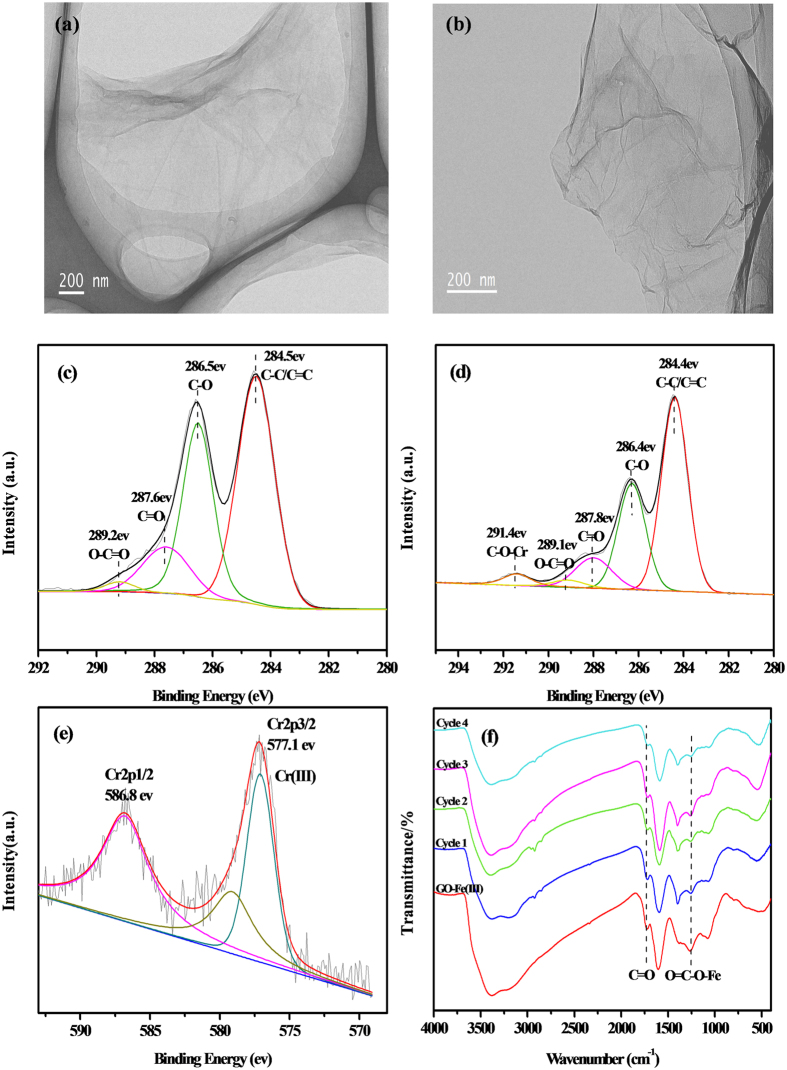
TEM images of the reduced products in (**a**) GO/Cr(VI) and (**b**) GO-Fe(III)/Cr(VI). C1s XPS spectra of (**c**) GO and (**d**) GO-Fe(III), and (**e**) Cr2p XPS spectra of GO-Fe(III) after the photoreduction of Cr(VI). FTIR of the reduced products of GO-Fe(III) with different reaction cycles (**f**).

To elucidate the photoreduction mechanisms of the GO-Fe(III) complex, the morphology and functional group variations of GO and GO-Fe(III) after the photoreaction were identified using TEM, XPS and FTIR. The TEM images (Fig. 5a,b) show that after photoreaction both GO and GO-Fe(III) were apparently aggregated with wrinkles which were tightly distributed on the basal planes of GO nanosheets to form grooved regions. And the surface of GO-Fe(III) was relatively rougher compared with that of GO because more oxygen containing functional groups on GO-Fe(III) were removed than GO, inducing stronger π-π interactions between the nanosheets.

The GO-Fe(III) complex maintained consistent reaction efficiency in 4 cycles of photoreduction (see supplementary Figure S6a), which could be attributed to the abundant carboxyl groups and the chemical stability of GO. On the other hand, supplementary Figure S6b shows the visible changes of the aqueous system after four cycles of photoreduction process. In comparison with the solution before the reaction, the color gradually changed from light yellow to black brown after the reaction, and as the number of repetitions increased, the solution darkened. These observations demonstrated that GO was gradually transformed to reduced graphene oxide (rGO) in black color because of the oxygen containing functional groups consuming on GO. Moreover, black precipitates were generated in the reaction solution, when it was leaved for overnight. This should be contributed by the aggregation and precipitation of rGO generated during the photoreduction.

The surface functional groups of GO after the reaction were analyzed using XPS. The C1s spectrum of GO (Fig. 5c) after the reaction exhibited identical peaks to that of GO before the reaction (Fig. 3e), indicating the oxygen-containing moieties were barely changed during the visible light irradiation. This phenomenon demonstrates that the optical electronic reaction of GO is slow and not obvious.

By contrast, the GO-Fe(III) reaction system after visible light irradiation, the C1s peak distribution (Fig. 5d) dramatically changed from the spectra before reaction (Fig. 3f), i.e., the carboxyl carbon in O–C = O decreased, and the carbon in C–O increased. The O/C ratio decreased, and the C–C/C = C content increased (Table 1), which indicates that the surface oxygen-containing functional groups of GO-Fe(III) were dramatically reduced in the photoreaction process.

Besides, a new peak shows up at 291.4 eV in Fig. 5d, which is assigned to the carbon in C–O–Cr(III). It seems the Cr^3+^ ions were coordinated in the precipitation. The Cr2p XPS spectrum of the GO-Fe(III)/Cr(VI) precipitate (Fig. 5e) shows a pair of peaks at 577.1 and 586.8 eV that correspond to Cr(III) 2p3/2 and Cr(III) 2p1/2^40^, respectively, further indicating the existence of Cr(III) in the precipitated rGO sheets. The elemental mapping of GO-Fe(III)/Cr(VI) after photoreaction also indicated the existence of chromium species (see supplementary Figure S7).

The FTIR spectra (Fig. 5f) further reveal the considerable changes in the various oxygen-containing functional groups on GO-Fe(III) after the reaction. In Fig. 5f, the peaks indicative of the carboxyl groups in C = O and the O = C–O–Fe both diminished after each reaction cycle, demonstrating that the oxygen-containing functional groups of GO-Fe(III) are gradually removed in the Fe(III)/Fe(II) cyclic reaction under visible light irradiation. In supplementary Figure S8, the C = O of the carboxyl groups on GO is converted into the epoxy C–O which is excited by light illumination. Simultaneously, the FTIR peak for the alkoxy in C–O–C was slightly decreased because the thermal stability of the alkoxy C–O–C on the surface is poor, and the alkoxy can be easily removed under light irradiation. In short, the GO sheets were reduced to rGO after the photoreaction with the existence of Fe^3+^. The generated rGO sheets adsorbed with Cr(III) spontaneously aggregated and precipitated to leave the aqueous solution.

### Influential Factors of Cr(VI) Photoreduction by the GO-Fe(III) Complex

**Figure 6:**
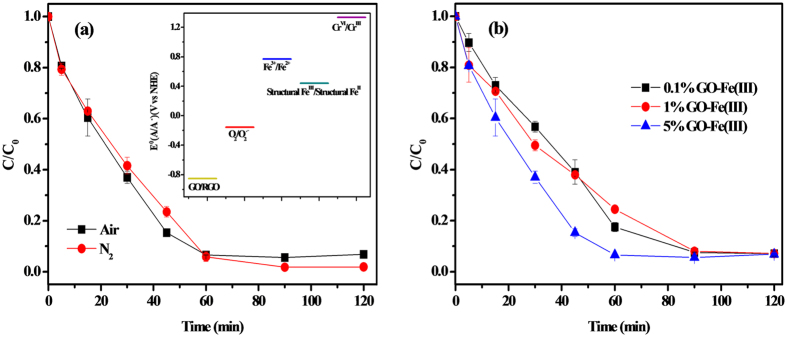
Effects of the dissolved O_2_ (**a**) and GO dose (**b**) with the presence of constant Fe(III) on the photoreduction of Cr(VI) by GO-Fe(III) under visible irradiation. The selected conditions are as follows: [Cr(VI)] = 40 μM, [GO] = 40 mg/L, [GO-Fe(III)]  = 40 mg/L, pH = 3. The reported reduction potentials of GO, oxygen and Fe species in this study are shown in (**a**). Note that the reduction potential of oxygen refers to that at the O_2_ concentration of 1 mol/L.

The effects of dissolved oxygen to the photoreduction of Cr(VI) by GO-Fe(III) were examined (Fig. 6a). The Cr(VI) reduction kinetics are similar in N_2_ and in an air-saturated solution, which indicates that oxygen is not competitive with Cr(VI) for accepting electrons. This observation can be readily explained from a thermodynamic perspective (see Fig. 6a). The excited GO (*E*^0^ (GO/ RGO) = −0.85 V vs NHE)^41^ preferentially donates an electron to the structural Fe(III) (*E*^0^ (structural Fe(III)/Fe(II)) = 0.44 V vs NHE)/surface Fe(III)/exchangeable Fe^3+^ (*E*^0^ (Fe^3+^/Fe^2+^) = 0.77 V vs NHE)^42^ instead of oxygen because of E(O_2_(1 M)/O_2_^.−^) = −0.155 V vs NHE)^43^. So the photo-induced electron would preferentially transfer to Fe(III) which has higher redox potential than O_2_. Then, the generated Fe(II) may reduce Cr(VI) to Cr(III). This observation indicates that when the GO-Fe(III) complex is under sunlight illumination, the reduction of Cr(VI) can be easily achieved, even under anaerobic conditions.

The effects of the GO proportion in the GO-Fe(III) complex on the photoreduction process were examined under visible light irradiation (Fig. 6b). The reaction rate constants of 5 wt% GO-Fe(III), 1 wt% GO-Fe(III), and 0.1 wt% GO-Fe(III) are 4.81 × 10^−2^ min^−1^ (R^2^ = 0.98), 2.87 × 10^−2^ min^−1^ (R^2^ = 0.97), and 2.81 × 10^−2^ min^−1^ (R^2^ = 0.98), respectively. These results implied that the increased content of GO can accelerate the electron transfer from GO-Fe(III) to Cr(VI). Although with lower GO loading, the time for complete reduction of Cr(VI) was lengthened to 90 minutes by the 0.1 wt% GO-Fe(III). On the other hand, the photoreduction of Cr(VI) was not obviously improved with the 5 wt% GO-Fe(III) complexes than the 1 wt% GO-Fe(III) complexes. This should be attributed to the aggregation of GO in high concentration and suggests that only the oxygen-containing functional groups at the edge of GO are effective sites for the photoreaction.

The Cr(VI) reduction rates at different pH values were shown in supplementary Figure S9a. Under acidic conditions, the reduction rate increased with decreasing pH value. The GO-Fe(III) complex is more stable at low pH; and with the pH increase, the ferric ions gradually generate precipitates. The removal rate of Cr(VI) is also affected by the chromium speciation which is pH-dependent. At low pH, the predominant Cr(VI) specie is HCrO_4_^−^, which shifts to CrO_4_^2−^ with the increase of solution pH. HCrO_4_^−^ and CrO_4_^2−^ display different reactivities^44^. The high removal efficiency at low pH is attributed to the highly protonated and positively charged surface of GO-Fe(III), which favors the approach of the negatively charged HCrO_4_^−^ via electrostatic attraction. Concurrently, the competition of OH^−^ with chromate ions reduces the Cr(VI) removal. The solution pH also affects the thermodynamic driving force of Cr(VI) photoreduction^45^,^46^. Therefore, the acid or faintly acid environment would be in favor of the Cr(VI) reduction.

The photoreduction was conducted with Cr(VI) in a serial of initial concentrations (see Supplementary Figure S9b). The photoreduction kinetics of Cr(VI) with an initial concentration of 20–100 μM well obeyed the first-order kinetic model with a good linear correlation (R^2^ > 0.9) (insert in Figure S7b). It has been reported that, at low Cr(VI) concentrations, the rate of Cr(VI) reduction is proportional to the initial Cr(VI) concentration^47^. At high Cr(VI) concentrations, the rate of Cr(VI) reduction begins to level off due to the site-saturation behavior caused by the precipitation of soluble solid such as the Cr(III) complex or the depletion of available Fe(II) from GO-Fe(III) in a heterogeneous reaction. Thus, the removal of Cr(VI) by a certain amount of the GO-Fe(III) complex under sunlight irradiation is faster in an aquatic environment containing less Cr(VI).

### The Mechanism of Cr(VI) Photoreduction and Removal by GO-Fe(III)

**Figure 7:**
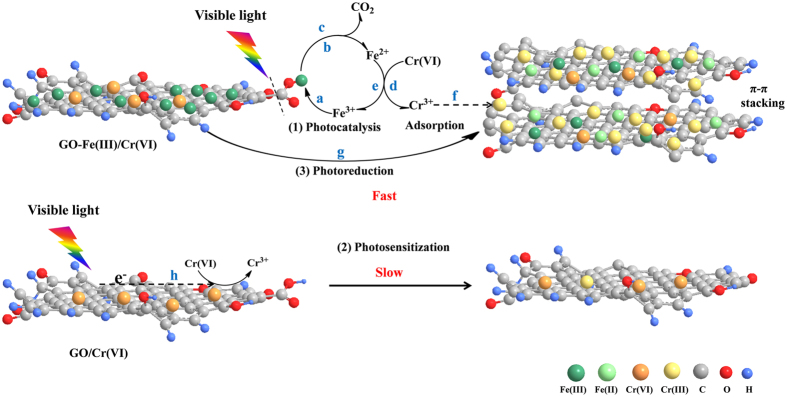
Proposed mechanisms for the photoreduction and adsorption of GO-Fe(III) to effectively remove Cr(VI) from the solution under visible light irradiation. (**a**) Fe^3+^ adsorption on GO; (**b**) Fe(III) transform to Fe(II); (**c**) decarboxylation; (**d**) Cr(VI) reduction; (**e**) Fe^2+^ oxidized by Cr(VI); (**f**) Cr^3+^ adsorption on rGO; (**g**) GO photo- transformed to rGO; (**h**) GO donate photo-electron to Cr(VI)).

Based on the photoreduction kinetics, influential factors, and structural characteristics before and after irradiation, the photoreduction mechanism and Cr(VI) removal by GO-Fe(III) are initially proposed in Fig. 7. The proposed mechanism is related to the synergistic interfacial effects of the oxygen-containing groups on GO and Fe(III), which include the photoreduction, adsorption and precipitation. First, Fe^3+^ is adsorbed by the carboxyl groups on the GO nanosheet surface to form the GO-Fe(III) complex (process a). Then, the photoreaction occurs at the solid-liquid interface, where the active sites of the Fe(III) complex with the surface carboxyl groups of GO, frequently converting Fe(III) to Fe(II) by GO-Fe(III) sensitization under visible light irradiation (process b), which is accompanied with the oxidative degradation of carboxyl groups to CO_2_ (process c). The generated Fe(II) can be present in the binding state on the surface of GO and the ionic state in the solution, all of which can be used to reduce Cr(VI) (process d). Concurrently, Fe^2+^ is restored to Fe^3+^ in the subsequent reduction reaction (process e), and the produced Cr^3+^ ions are adsorbed by GO or rGO (process f), which decreases the total chromium concentration of the solution. The photoreaction mechanism of GO-Fe(III) is similar to that of ubiquitous complexes of dissolved organic matter (DOM) with Fe(III) (i.e., DOM-Fe(III)) in a natural environment^35^,^36^,^48^, but the environmental behavior of GO-Fe(III) is different from the interfacial photoreaction of Fe(III)/Fe(II) and Cr(VI)/Cr(III) since the GO sheets are also photoreduced to rGO (process g). The enhanced π-π attraction between layers and Cr(III) adsorption further promote the aggregation, resulting co-precipitation of rGO and Cr(III). On the other hand, GO itself has slight photosensitization because of the quinonyl groups^49^,^50^, which could slowly transfer the photo-electron to the adsorbed Cr(VI) realizing its minor reduction (process h).

Presumably, in a complicated aqueous environment containing GO nanosheets, Fe(III) and highly toxic Cr(VI), the GO sheets may coordinate with Fe(III) to form complex which will reduce Cr(VI) to less toxic positively charged Cr^3+^ ions and simultaneously be converted to rGO under visible light irradiation. Subsequently, the rGO sheets will adsorb Cr^3+^ ions and spontaneously aggregate to form precipitations. In short, both GO and toxic Cr(VI) can be removed from the aqueous environment with the existence of iron ions as the catalyst under light irradiation.

## Conclusions

The widespread applications of GO in diverse fields opens the possibility for GO to inevitably enter into natural aquatic environments. The environmental factors (such as light, transition metals, heavy metals, dissolved oxygen, and pH) would influence the transportation and transformation of GO in the environment. Our work suggests that GO will form photoactive complex with Fe(III) in the aqueous environment. The GO-Fe(III) complex may effectively reduce Cr(VI) to Cr(III) under visible light irradiation because of the decarboxylation on GO. The losing of carboxyl groups on GO generates rGO which is also able to adsorb Cr(III) but easier to aggregate and precipitate. The transformation of GO with the help of iron ions under visible light indicated a promising route to convert and remove the highly toxic Cr(VI) pollutant from the aqueous environment.

## Methods

### Chemicals

Graphite (325 mesh, 99.8%, Alfa Aesar), K_2_Cr_2_O_7_ (Aldrich), FeCl_3_ (Aldrich), 1,10-phenanthroline monohydrate (AR, Sigma-Aldrich, 98%), and diphenylcarbazide (DPC) (Aladdin, HPLC, >98%) were used in the experiment. All other reagents were of analytical grade.

Graphene oxide (GO) was synthesized from graphite flakes using the modified Hummers method^51^. The GO-Fe(III) complex was prepared by mixing the GO solution (pH = 2, adjusted by HCl) with FeCl_3_ (1 mol/L) under stirring for 24 h. Then, the mixed solution was centrifuged at 10000 rpm and washed with deionized water several times until the upper solution contained no ferric ions. The resultant precipitate was dispersed into water as photoreaction materials and dried at 60 °C for structural characterization. Different GO-loading complexes were synthesized by changing the ratio of GO to 0.1 wt%, 1 wt% and 5 wt%.

### Photoreduction of Cr(VI) by GO-Fe(III) Complex

For a typical photoreaction, GO-Fe(III) and GO were suspended in 50 mL aqueous solution. The selected initial concentrations of GO-Fe(III), GO and Cr(VI) were 40 mg/L, 40 mg/L, and 40 μM, respectively. The reaction solutions were always freshly prepared at an initial pH of 3.00 ± 0.05, except for experiments that considered the effect of pH values. The initial pH was adjusted using diluted HCl and NaOH. Before illumination, GO/Cr(VI) and GO-Fe(III)/Cr(VI) were stirred for 30 min in the dark to obtain the adsorption/desorption equilibrium. Deaerated suspensions were prepared by purging nitrogen gas for at least 30 min before irradiation and throughout the entire experimental process. At regular time intervals, approximately 2 mL of the reaction solutions were sampled and filtered through a 0.22 μm filter for further detection. Duplicate runs were performed for each experiment. All experiments were conducted in a 60 mL Pyrex vessel with magnetic stirring. The irradiation source was a 500 W halogen lamp with a UV cutoff filter (λ > 420 nm) in a XPA-7 type photochemical reactor (Xujiang Electromechanical Plant, Nanjing, China). The temperature was maintained at room temperature by circulating cooling water.

The Cr(VI) concentration was determined using the diphenylcarbazide (DPC) method at a wavelength of 540 nm in a Shimadzu UV-2550 spectrophotometer^52^. Briefly, a 1 mL sample was added to 1 mL of buffer solution (V(H_2_SO_4_):V(H_3_PO_4_):V(H_2_O) = 1:1:2), and 2 mL chromogenic reagent (0.2 g diphenylcarbazide mixture with 100 mL acetone solution V (acetone:H_2_O) = 1:1) was subsequently added. To prevent Fe(II) from interfering with the determination of the Cr(VI) concentration, the Cr(VI) concentration was calibrated using a blank control. Dissolved Fe^2+^ was quantified using a 1,10-phenanthroline method at a wavelength of 510 nm in a Shimadzu UV-2550 spectrophotometer^53^. Briefly, the premixture was prepared by combining 0.5 mL of sodium acetate/acetic acid buffer (pH = 5.7), 0.25 mL of ammonium fluoride solution (0.4 mol/L), 0.25 mL of 1,10-phenanthroline solution (20 mmol/L), and 1 mL of sample solution. The Fe(II) molar concentration (μmol/L) was calculated as follows: [Fe(II)] = (2A_i1 _− A_i2_)/0.011, where A_i1_ is the absorbance of the sample after a chromogenic reagent was added at 510 nm and A_i2_ is the absorbance of the sample at 510 nm. The Cr(VI) reduction efficiency at a given time (*t*) was calculated as follows: reduction efficiency (%) = (*C*_0_ − *C*_t_)/*C*_0_ × 100%. A pseudo first-order model was applied to describe the kinetics of Cr(VI) photoreduction by GO-Fe(III). The first-order constant *k* (min^−1^) was determined according to the following equation: ln(*C*_0_/*C*_t_) = *kt*, where *C*_t_ is the concentration of Cr(VI) at time t, *C*_0_ is the initial concentration of Cr(VI), *k* is the reduction rate constant, and *t* is the irradiation time. The total Cr and Fe concentrations in the supernatant were determined using atomic absorption spectrometry (Perkin Elmer Analyst 700). The variation of the surface functional groups and morphologies of the GO-Fe(III) complex were observed using XPS, FTIR and TEM.

### Characterization of GO-Fe(III)

The surface morphologies of GO and GO-Fe(III) were characterized using SU-8000 scanning electron microscopy (SEM) (Hitachi, Tokyo) and FEI Tecnai G^2^ F20 S-TWIN Transmission Electron Microscopy (TEM) (FEI, America). Thermogravimetric analyses (TGA) was conducted on a SDT Q600 V8.2 Build 100 apparatus at a heating rate of 5 °C min^−1^ from room temperature to 850 °C in air and N_2_ flow. Atomic force microscopy (AFM) images of GO and GO-Fe(III) on a freshly cleaved mica surface were obtained using a Nanoscope III in tapping mode with a NSC14/no Al probe (Dimension Icon, Veeco). After sonication was performed for 5 min, a droplet of the sample dispersion (~0.01 mg/mL) was cast onto a freshly cleaved mica surface. The sample was maintained at room temperature overnight to allow evaporation of the water. The surface functional groups were observed by Fourier transform infrared spectroscopy (FTIR) and X-ray photoelectron spectroscopy (XPS). The FTIR spectra were recorded in the 4000–400 cm^−1^ region with a resolution of 4 cm^−1^ using a Bruker Vector 22 FTIR spectrometer. The XPS experiments were performed on an Escalab 250 Xi with a resolution below 0.5 eV, and the C1s, Fe2p and Cr2p peak spectra were analyzed using XPS Peak 4.1 software.

## Additional Information

**How to cite this article**: Liu, R. *et al*. A New Insight of Graphene oxide-Fe(III) Complex Photochemical Behaviors under Visible Light Irradiation. *Sci. Rep.* **7**, 40711; doi: 10.1038/srep40711 (2017).

**Publisher's note:** Springer Nature remains neutral with regard to jurisdictional claims in published maps and institutional affiliations.

## Supplementary Material

Supplementary Information

## Figures and Tables

**Table 1:** Elemental compositions on the surface of GO and GO-Fe(III) before and after photoreaction with Cr (VI).

Sample	O/C	%C-C/C = C	%C-O	%C = O	%O-C = O
GO	0.46	52.12	23.94	20.71	3.22
GO/Cr(VI)	0.45	50.66	32.15	13.47	3.72
GO-Fe(III)	0.47	50.17	25.56	20.62	3.56
GO-Fe(III)/Cr(VI)	0.39	56.69	28.46	11.81	3.14
