# Supplementary material for: A New Insight of Graphene oxide-Fe(III) Complex Photochemical Behaviors under Visible Light Irradiation
Source: Sci Rep. 2017 Jan 13;7:40711. doi: 10.1038/srep40711 (PMC5234028; doi:10.1038/srep40711)
Supplement: Supplementary Information [file srep40711-s1.pdf]

Supplementary Information for *Scientific Reports*

A New Insight of Graphene oxide-Fe(III) Complex Photochemical Behaviors under Visible Light Irradiation

Renlan Liu[1,2], Xiaoying Zhu[1,2], Baoliang Chen[1,2, *]

1. Department of Environmental Science, Zhejiang University, Hangzhou, Zhejiang 310058, China;

2. Zhejiang Provincial Key Laboratory of Organic Pollutant Process and Control, Zhejiang University

Corresponding author

E-mail: blchen@zju.edu.cn (B. Chen)

Tel: 86-571-8898-2587

Fax: 86-571-8898-2587

Supporting information consists of 8 pages, including this one. There are 9 Figures.

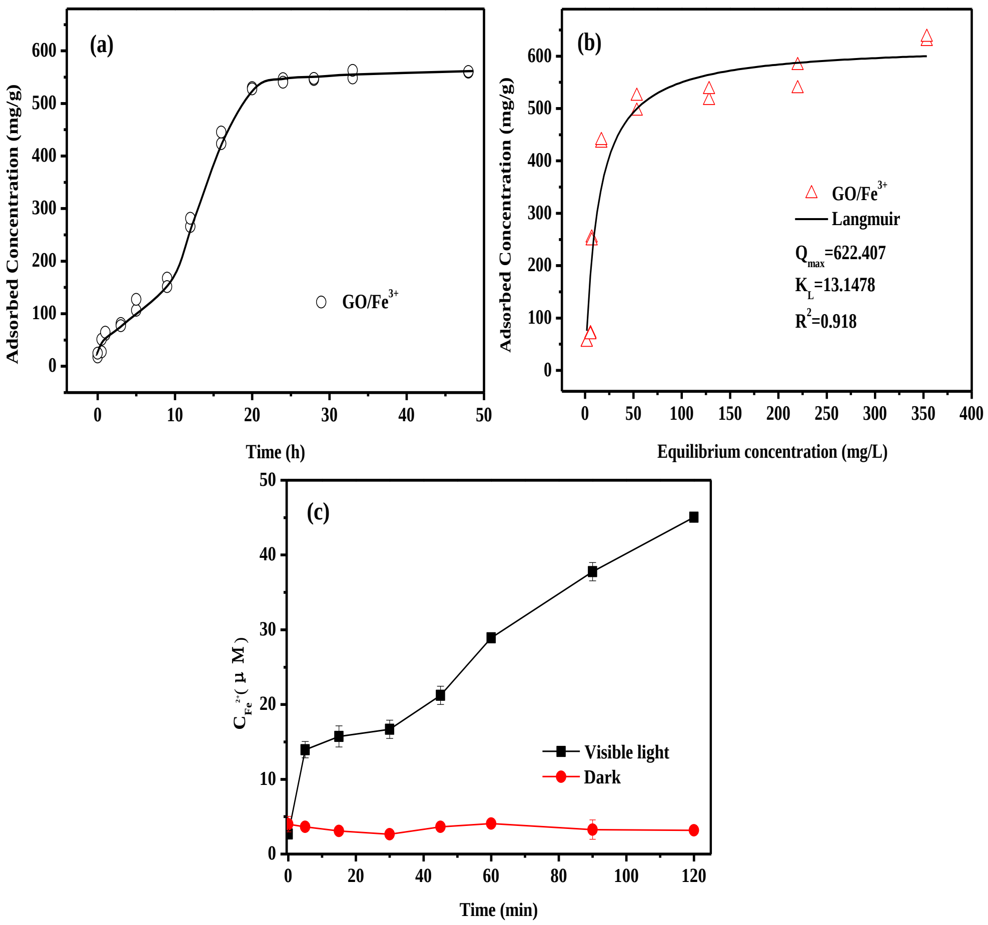

Figure S1. Adsorption kinetics of Fe^{3+} on GO (The initial concentration of Fe^{3+} was 80 mg/L, GO was 1 mg) (a). Adsorption isotherm of Fe^{3+} on GO. Solid lines represent isotherm fitted by the Langmuir model (b). The variation of Fe^{2+} ions in the GO-Fe(III)/Cr(VI) system in the dark and under visible light irradiation (c).

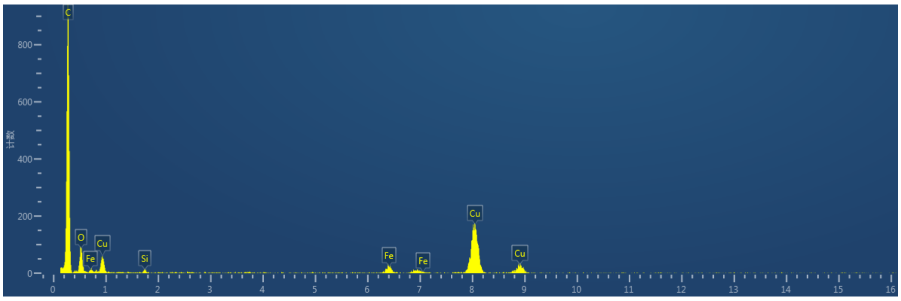

Figure S2. EDX analysis of the GO-Fe(III) complex. The EDX analysis of the GO-Fe(III) complex shows the dominant elements including carbon (C), oxygen (O) and iron (Fe) which indicates the iron ions have successfully combined on GO.

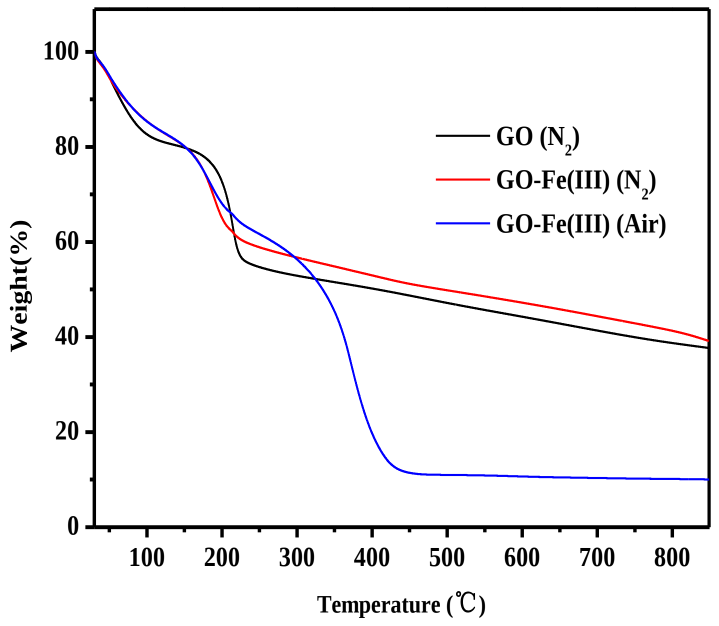

Figure S3. TG curves of GO and GO-Fe(III) in the nitrogen atmosphere and GO-Fe(III) in air.

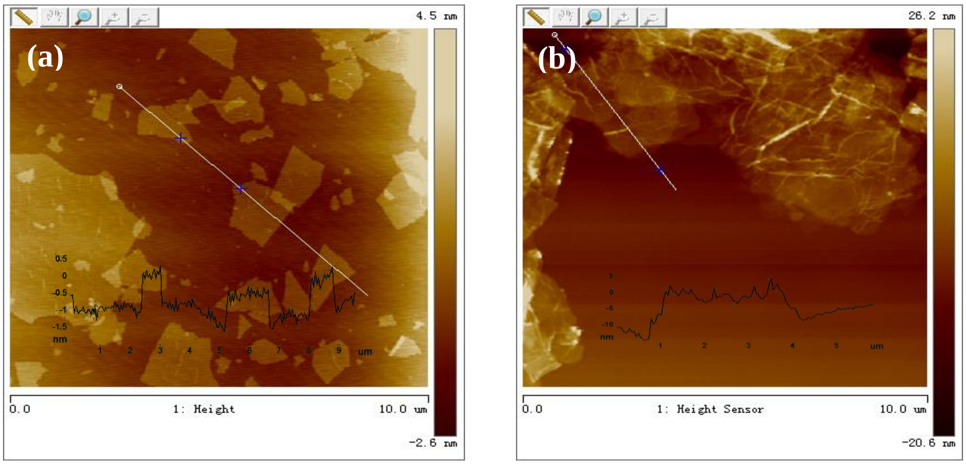

Figure S4. AFM images of GO (a) and GO-Fe(III) (b).

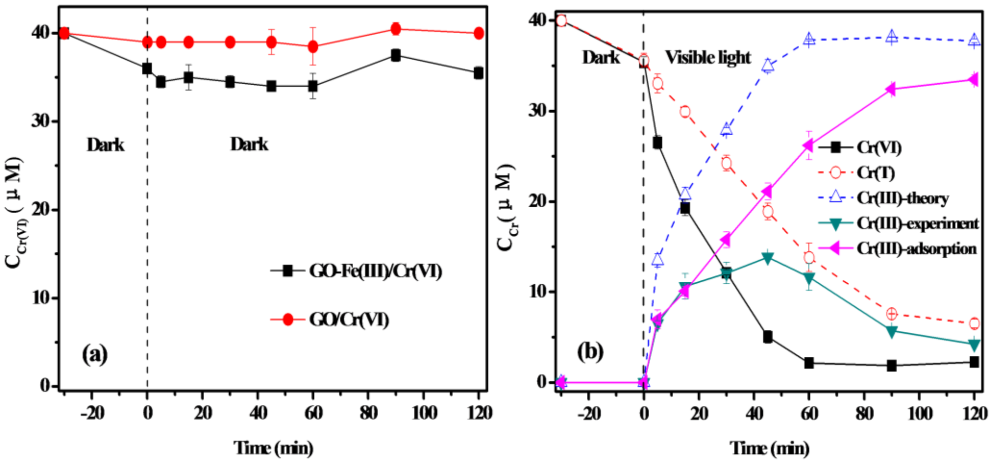

Figure S5. The Cr(VI) concentration variation in the GO-Fe(III)/Cr(VI) and GO/Cr(VI) systems in the dark (a). The variation of Cr(T), Cr(VI), Cr(III) in the solution and Cr(III) adsorption on GO-Fe(III) under visible light irradiation (b).

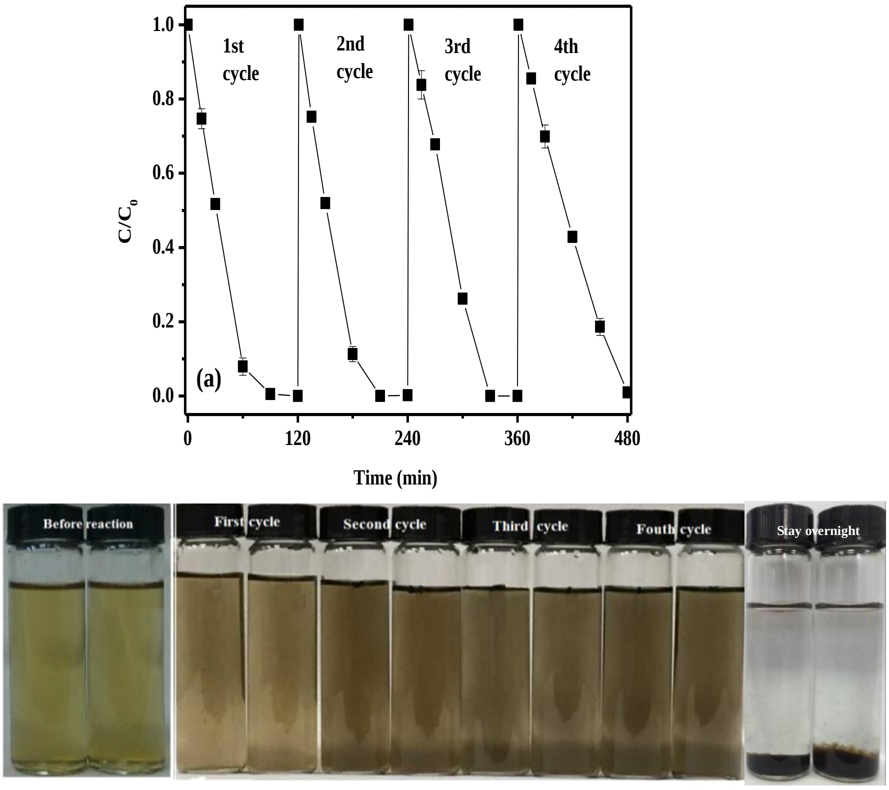

(b)

Figure S6. Catalytic performance in the cyclic reduction of Cr(VI) in the GO-Fe(III) system under visible-light illumination (a). Photograph of GO-Fe(III) before reaction, after every cycle and stay overnight (b), conditions: GO-Fe(III)=40 mg/L; pH=3, Cr(VI)=40 μM run^{-1}, reaction time=120 min run^{-1}. After four cycling runs, GO-Fe(III) retained good activity for the photoreduction of Cr(VI). After Fe^{3+} adsorption on GO, the GO-Fe(III) can also act as a role of photocatalyst in the process of Fe(III)/Fe(II) and Cr(VI)/Cr(III) cycle.

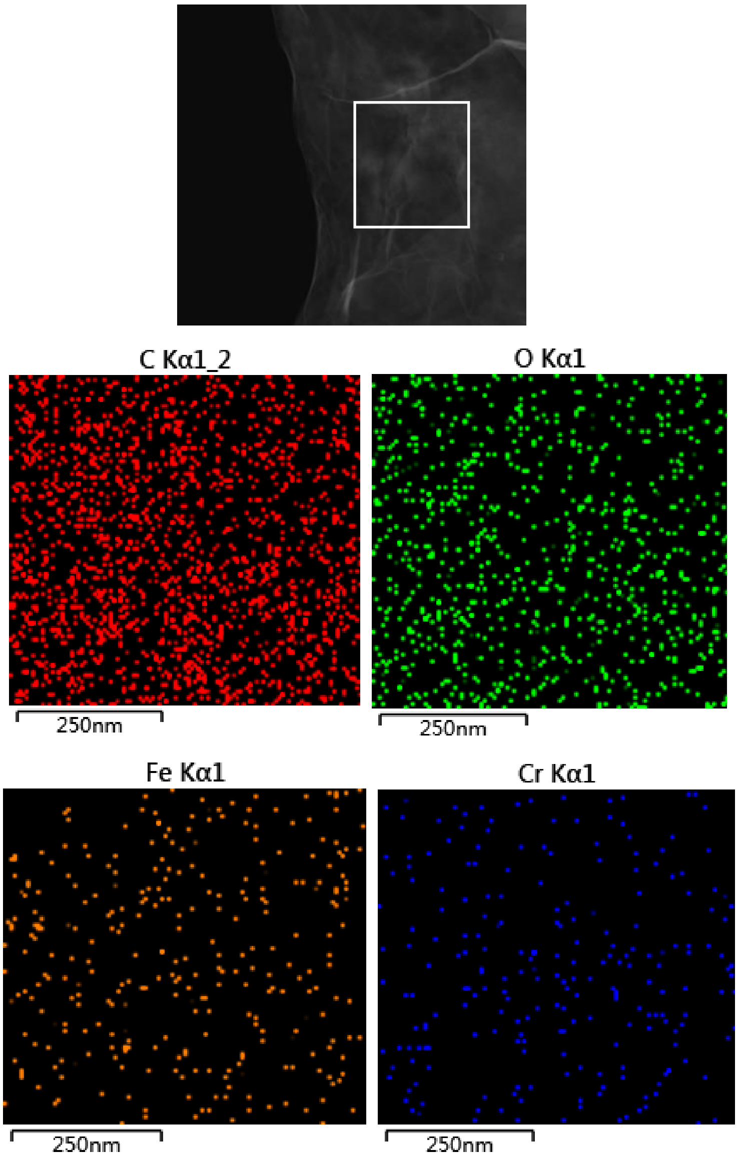

Figure S7. Elemental mapping of GO-Fe(III)/Cr(VI) after reaction.

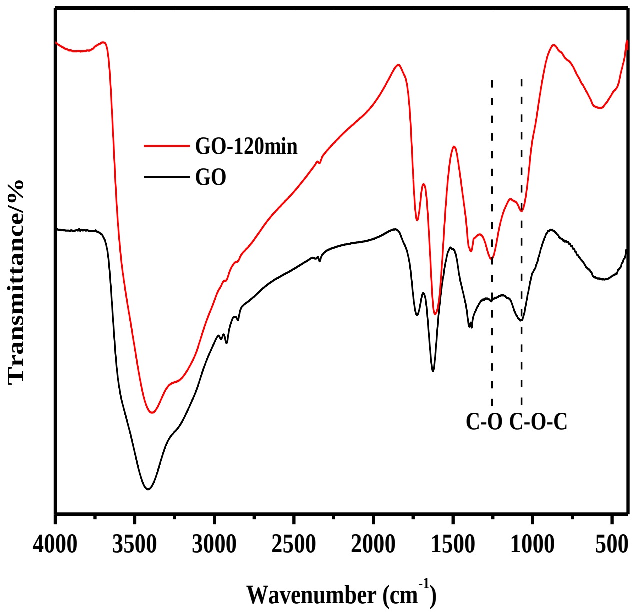

Figure S8. FTIR of the reduced products of GO after the photoreaction. The FTIR peak for the alkoxy in C–O–C was slightly reduced because the thermal stability of alkoxy C–O on the surface is poor, and easily removed under light irradiation. And the C=O of the carboxyl groups on GO is converted into the epoxy C–O which is excited by light illumination.

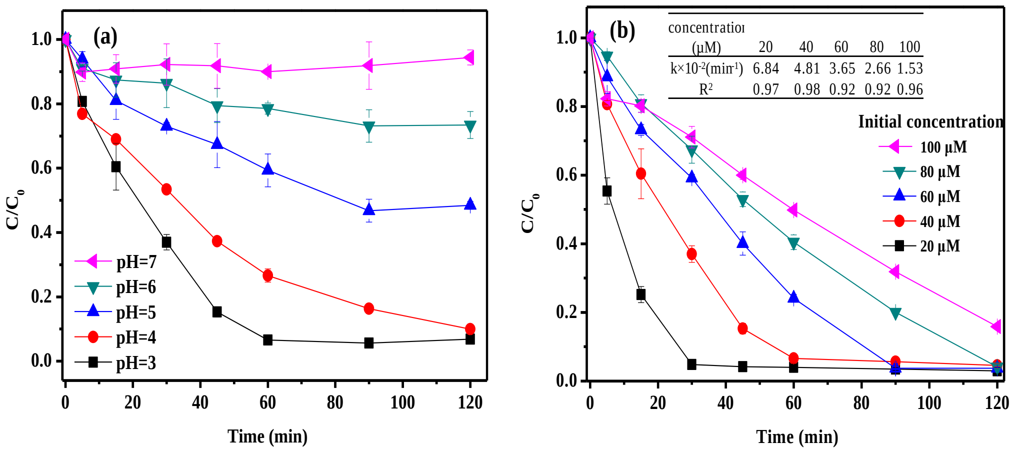

Figure S9. Effects of the initial (a) pH, (b) Cr(VI) concentration with the presence of constant Fe(III) on the photoreduction of Cr(VI) by GO-Fe(III) under visible irradiation. The selected conditions are as follows: [Cr(VI)]=40 μM, [GO] =40 mg/L, [GO-Fe(III)] =40 mg/L, pH=3.
